# Unraveling Negative Expectations and Nocebo-Related Effects in Musculoskeletal Pain

**DOI:** 10.3389/fpsyg.2022.789377

**Published:** 2022-03-16

**Authors:** Giacomo Rossettini, Andrea Colombi, Elisa Carlino, Mattia Manoni, Mattia Mirandola, Andrea Polli, Eleonora Maria Camerone, Marco Testa

**Affiliations:** ^1^Department of Neuroscience, Rehabilitation, Ophthalmology, Genetics, Maternal and Child Health, University of Genova, Genova, Italy; ^2^School of Physiotherapy, University of Verona, Verona, Italy; ^3^Department of Neuroscience, University of Turin Medical School, Turin, Italy; ^4^Pain in Motion (PAIN) Department, Vrije Universiteit Brussel, Brussels, Belgium; ^5^Department of Public Health and Primary Care, KU Leuven, Leuven, Belgium; ^6^Research Foundation, Flanders (FWO) Postdoctoral Fellow, Brussels, Belgium; ^7^Department of Psychology, University of Milan-Bicocca, Milano, Italy

**Keywords:** nocebo effects, contextual factors, pain, musculoskeletal, physiotherapy, expectation, predictive brain, placebo effects

## Abstract

This Perspective adapts the ViolEx Model, a framework validated in several clinical conditions, to better understand the role of expectations in the recovery and/or maintenance of musculoskeletal (MSK) pain. Here, particular attention is given to the condition in which dysfunctional expectations are maintained despite no longer being supported by confirmatory evidence (i.e., belief—lifting the arm leads to permanent tendon damage; evidence—after the patient lifts the arm no tendon damage occurs). While the ViolEx Model suggests that cognitive immunization strategies are responsible for the maintenance of dysfunctional expectations, we suggest that such phenomenon can also be understood from a Bayesian Brain perspective, according to which the level of precision of the priors (i.e., expectations) is the determinant factor accounting for the extent of priors’ updating (i.e., we merge the two frameworks, suggesting that highly precise prior can lead to cognitive immunization responses). Importantly, this Perspective translates the theory behind these two frameworks into clinical suggestions. Precisely, it is argued that different strategies should be implemented when treating MSK pain patients, depending on the nature of their expectations (i.e., positive or negative and the level of their precision).

## Introduction

Musculoskeletal (MSK) pain is ranked at the top of non-communicable diseases (Safiri et al., 2021), representing a profound burden for all socioeconomic and healthcare systems worldwide (Briggs et al., 2020). Although most MSK pain states have a good prognosis, there is a substantial proportion of patients who do not show spontaneous remission or do not respond favorably to first-line interventions and usual care, thus developing long-lasting symptoms, disabilities, and participation loss (Blyth et al., 2019).

The management of these patients is challenging because their subjective complaints (i.e., level of disability) rarely correlate with clinical and radiological findings (i.e., structural impairments; Rondoni et al., 2017; Tonosu et al., 2017; Viceconti et al., 2020). Thus, the lack of an identifiable pathology observed in various MSK diseases (i.e., low back pain and fibromyalgia) can have clinical implications. On the one hand, patients may repetitively seek care, thus adopting unhelpful health-seeking behaviors. For example, they may contact various health care providers (Ng et al., 2020), overuse health services (Sajid et al., 2021), request complementary and alternative medicine (Setchell et al., 2021), and misuse drugs (Ashaye et al., 2018). On the other, clinicians risk to invalidate patients’ experience (De Ruddere et al., 2013), offer contradictory explanations about their condition (Bunzli et al., 2013; Mannion et al., 2013), and generic diagnoses (Yunus, 2007). As a result, patients often experience negative emotions and adopt unhelpful coping strategies (i.e., catastrophic thinking, avoidance of movement), which are, *per se*, capable of worsening their clinical conditions and foster symptoms persistence (Bunzli et al., 2015; Darlow, 2016). Moreover, they may develop negative expectations about the course of their illness and the likely outcomes (Kravvariti et al., 2018, 2021; Thomaidou et al., 2021).

Negative expectations impact MSK pain (Hallegraeff et al., 2012; Geurts et al., 2017; Hayden et al., 2019; Fishbain and Pulikal, 2020; Mohamed et al., 2020), playing a significant role in transitioning from acute to persistent pain (Manai et al., 2019), and maintaining symptoms (Blasini et al., 2017; Klinger et al., 2017). Moreover, they can bias symptom perception (Handley et al., 2013; Bagaric et al., 2021) and reduce treatment effectiveness (Colloca et al., 2018; Corsi et al., 2019), inducing nocebo-related effects (Petrie and Rief, 2019; Benedetti et al., 2020). Within the MSK context, nocebo-related effects refer to those negative responses that follow treatment administration (i.e., painkillers, manual therapy, and therapeutic exercises) associated with a negative expectation (Rossettini et al., 2020a). Investigating patients’ beliefs and expectations represent a priority for clinicians treating MSK pain (Lewis and O'Sullivan, 2018; Caneiro et al., 2020; Lin et al., 2020; Cook et al., 2021; Hutting et al., 2021; Lewis et al., 2021). However, such practice is not routinely implemented in clinical practice (Rossettini et al., 2019, 2020c). As emerged in previous surveys, clinicians involved in MSK care report difficulties in managing patients’ expectations and avoid nocebo-related effects (Palese et al., 2019a; Cadorin et al., 2020; Rossettini et al., 2020b; Bisconti et al., 2021). This lack highlights the need for clinicians to have a framework that they can apply in everyday practice.

This Perspective has two aims. First, to provide clinicians with a better understanding of why some MSK patients hold on to their negative expectations. To this end, we will adapt the ViolEx Model (Kube et al., 2020) and the Bayesian brain hypothesis (Büchel et al., 2014). Second, we suggest some key strategies that clinicians can use to help patients update their dysfunctional expectations based on the theoretical frameworks discussed.

## The ViolEx Model and the Bayesian Brain

### The ViolEx Model

**Figure 1:**
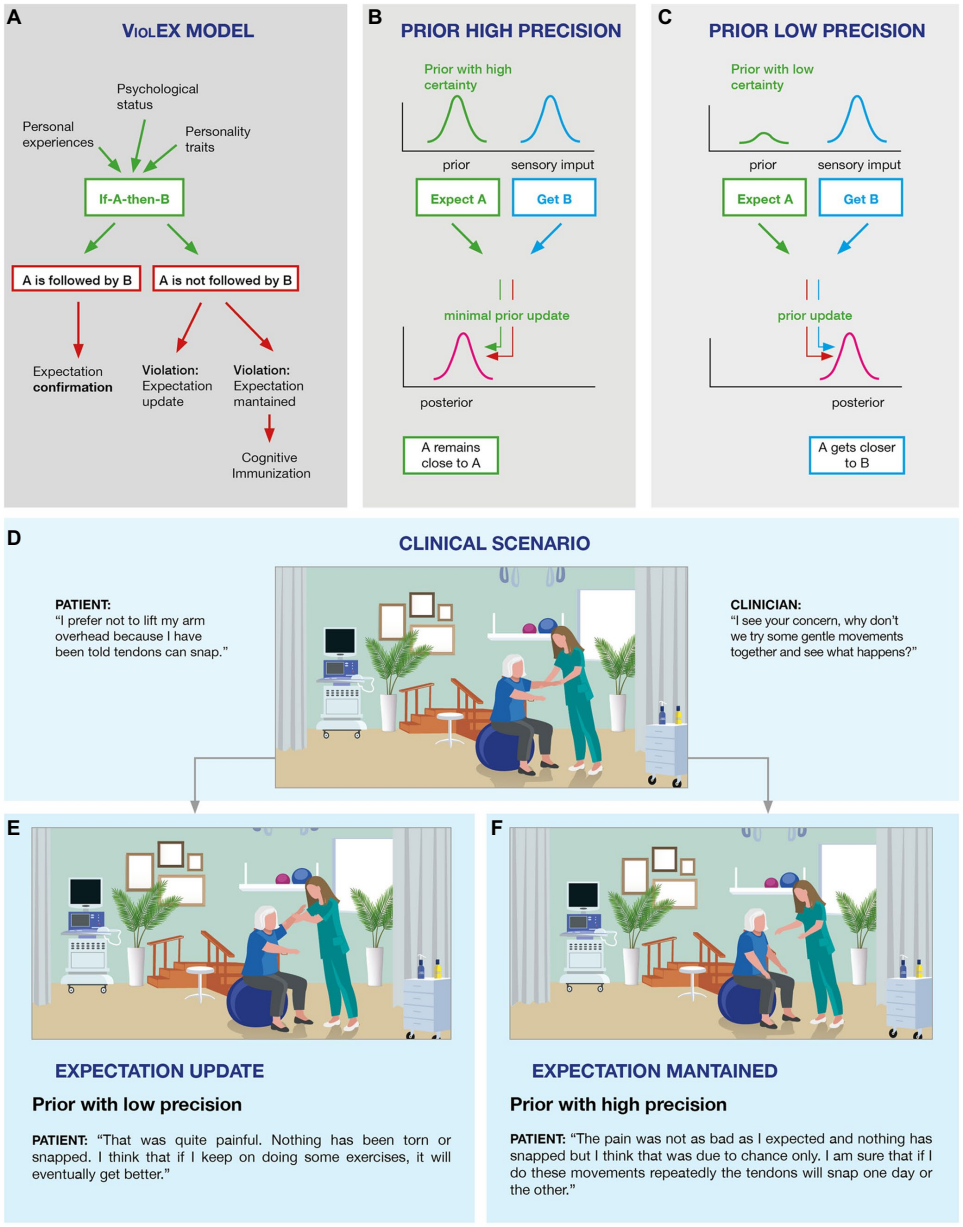
The ViolEx Model and the Bayesian Brain: from theory to clinical practice in musculoskeletal (MSK) pain. The ViolEx Model **(A)** and the Bayesian brain **(B,C)**. Image **(A)** is a schematization of the ViolEx Model showing different outcomes depending on whether the violation of expectations is followed by immunization or change, resulting in either expectations maintenance or expectations updating, respectively. Image **(B)** is an example in which a prior with a high level of certainty is considered reliable and therefore undergoes minimal updating, while the interpretation of the sensory data is shifted toward the prior, resulting in a biased percept. Image **(C)** is an example in which a prior with a low level of certainty is updated to better fit sensory data, resulting in a posterior which is a better proxy of the sensory information. Examples of clinical scenarios **(D–F)**. Image **(D)** shows a typical clinical situation in which the clinician asks the patient with MSK pain to perform a basic exercise (lift the arm), while the patient is reluctant to do it due to their negative expectations (i.e., pain/injury). The patient finally agrees and lifts the arm, without facing negative consequences. Such situation may result in two different outcomes: in **(E)** the positive experience associated with the exercise leads to the violation of the patient’s negative expectation with an update (low prior); whereas in **(F)** the positive experience associated with the exercise is not sufficient to violate the patient’s negative expectation, thus maintaining the previous experience (high prior).

When patients receive a MSK treatment, they can either “get what they expect” or “not get what they expect.” The ViolEx Model offers an interesting description of the possible outcomes and consequences of such expectations match/mismatch (Kube et al., 2020). The starting point of this model is that individuals develop expectations that are based on their own experiences: if a patient with neck pain has a negative past experience with therapeutic exercises, it is likely that this patient will have negative expectations regarding such treatment in the future. Moreover, expectations are also shaped by personality traits: that is, neuroticism, pessimism, and trait anxiety have been associated with a tendency to expect worse outcomes in situations perceived as threatening (Barlow et al., 2014). On the whole, these expectations produce an internal model of “if A-then-B,” that is “*if I do this exercise (A) then I will experience side-effects (B)*.” When the internal model is consolidated, three different scenarios can occur. In the first scenario, reality matches the internal model and expectations are confirmed and reinforced: it means that the patient performs the exercise, the exercise produces side effects leading to a consolidation and reinforcement of negative expectations. The consequence is that the patient will learn that the treatment produces negative effects and he/she will therefore seek a different intervention in the future (Figure 1A; *expectation confirmation*).

In the second scenario, reality does not match the internal model and expectations are violated: it means that the patient performs the exercise and does not experience negative side effects. Thus, two possible outcomes can occur: expectations can be updated, based on the newly acquired information (i.e., the patient experiences that the prior negative expectations toward the exercise was wrong and learns to no longer be worried about such treatment; Figure 1A; *expectation violation followed by an update*), or they can be maintained, despite the disproving evidence (i.e., despite treatment intake is not followed by the predicted negative consequences, the patient persists in believing that the exercise is likely to be followed by negative side effects; Figure 1A; *expectation violation followed by dysfunctional beliefs maintenance*).

Out of these three scenarios, the third is the problematic one. The ViolEx Model explains this last scenario introducing the concept of “*cognitive immunization*,” which indicates the engagement of strategies adapted to reappraise new information in such a way that the discrepancy between the evidence and the prior expectation is reduced, contributing to the maintenance of negative beliefs, despite the occurrence of conflicting events (Rief and Petrie, 2016; Rief and Joormann, 2019; Kube et al., 2019b).

Recently, the ViolEx Model has given interesting insights into clinical conditions such as depression (Kube et al., 2017; Rief and Joormann, 2019). Precisely, patients suffering from depression have been shown to maintain negative beliefs, even when presented with positive evidence disconfirming their prior negative beliefs (i.e., the example of Figure 1A; expectation violation followed by dysfunctional beliefs maintenance; Korn et al., 2014; Liknaitzky et al., 2017; Everaert et al., 2018). Interestingly, Kube et al. (2019a,b) have successfully demonstrated that by delivering instructions either promoting or discouraging cognitive immunization, it was possible to enhance negative expectations maintenance or to reduce them, respectively, demonstrating that cognitive immunization is likely to underlie dysfunctional expectations maintenance. Another interesting finding is that healthy individuals have been shown to have a positive bias, meaning that they are less likely to update positive priors if presented with contradictory negative evidence (Sharot et al., 2007, 2011, 2012; Garrett and Sharot, 2017), yet, such bias is abolished in the presence of perceived threat, in which case they become more responsive to the newly acquired negative evidence, updating their expectations (Sharot et al., 2007, 2011, 2012; Garrett and Sharot, 2017). This could be an important finding given that MSK pain patients report high threat perception linked to their experience of pain (Ochsner et al., 2006; Turk and Wilson, 2010); accordingly, this could indicate that MSK pain patients (similarly to depressed ones), become more susceptible to negative evidence, promoting the maintenance of dysfunctional expectations. Accordingly, it has been shown that patients with somatization syndrome (Rief et al., 2006) and MSK pain (Traeger et al., 2019; Barbari et al., 2021; Barth et al., 2021; Cashin et al., 2021; Cheung and Soundy, 2021; Jones et al., 2021) do not often use positive reassurance and education to update their negative dysfunctional beliefs and expectations.

Overall, previous research has shown the ViolEx model to be a valuable framework to better understand dysfunctional expectations maintenance in some clinical populations; therefore, we suggest that such model should be used to understand pain in MSK patients. In the daily practice clinicians often see MSK patients maintaining their dysfunctional expectations even if positive and reassuring evidence are provided (i.e., ViolEx Model; Figure 1A, scenario three). A good example is the case of patients that expect that their shoulder would break if they lift their arm (Figure 1D). When patients manage to fully lift their arm under the clinician supervision, and realize that their shoulder does not snap, the positive scenario (interiorizing that they can lift their arm without any negative consequences; Figure 1E) is less likely than the negative one represented by the “cognitive immunization” strategies (interpreting the event as lucky or as an exception to the rule; Figure 1F). Although this model is yet to be empirically tested upon MSK patients, it is likely to suggest that dysfunctional expectations maintenance and cognitive immunization strategies are recurrent in this clinical population.

Although cognitive immunization explains why dysfunctional expectations are maintained, it is yet to be understood why some patients implement such strategies to protect their negative beliefs and why others do not, updating their negative expectations with new positive evidence. We suggest that such differences in updating responses can be understood, at least to some extent, from a Bayesian perspective.

### The ViolEx Model From a Bayesian Perspective

From a Bayesian perspective, our brain is conceptualized as a predictor machine that generates predictions, known as *priors*, about the expected sensory inputs. The integration between the prior and the sensory input results in a posterior (the percept), which can be more or less influenced by the prior and by the sensory data, depending on their level of precision (i.e., data encoded as probabilistic representatations; Friston, 2008, 2010; Büchel et al., 2014; Seymour and Mancini, 2020). Within this framework, a prior with high precision is considered reliable and, therefore, will exert greater influence on the interpretation of the incoming sensory input, resulting in a posterior (i.e., percept) which is biased toward the prior (Figure 1B). Differently, a prior with low precision will be considered unreliable, and therefore, will be given less consideration when interpreting the incoming sensory input, resulting in a posterior (i.e., percept) that is a better proxy of the sensory data (Figure 1C). Consider a sensory input which does not match the prior; if the prior has higher precision this is likely to result in a smaller prediction error (PE) (i.e., since the percept is biased toward the prior, there will be less discrepancy between the prior and the percept), compared to a prior with less precision (i.e., since the percept is a better proxy of the sensory information there will be more discrepancy between the prior and the percept), resulting in greater prior updating in the latter case (Friston, 2008, 2010; Büchel et al., 2014; Seymour and Mancini, 2020).

With this Bayesian model in mind, it is possible to better understand the second and third scenarios of the ViolEx Model discussed in the previous section (Figure 1A; Kube et al., 2020). Precisely, expectation violation followed by update could be attributed to one’s having a prior with low precision which is updated accordingly with the newly acquired evidence (i.e., according to this view, the patient who is shown that lifting their arm does not lead to their shoulder to break and therefore updates such dysfunctional belief does not have a highly confident negative prior).

Differently, expectation violation followed by the maintenance of the dysfunctional belief would be understood as the consequence of one’s having a highly precise prior which is considered highly reliable, and therefore, the newly acquired disconfirmatory evidence is not sufficient to disproof and update such strong prior. Indeed, the attribution of high certainty to such prior can motivate the engagement of higher-order cognitive strategies, such as cognitive immunization (Kube et al., 2017). For example, MSK patients that, during a clinical session, manage to lift their arm without any negative consequences to their shoulder but still belief that lifting the arm will eventually lead to their shoulder to break, are likely to have a highly precise prior and might discard the positive evidence (success in lifting the arm) which might be classified as an exception instead of the rule (example of cognitive immunization; Figure 1F).

As we have suggested here, the Bayesian framework can give further insights into the mechanisms of expectations updating described by the ViolEx Model. Yet, it is important to highlight that these two accounts differ in one major aspect. While the ViolEx model is cognitivist in nature; that is, it is premised on the existence of cognitive states called “expectations” and is concerned with the relation between expectations and symptoms independently of discussions of neuronal processes (Kube et al., 2020), the Bayesian brain hypothesis (also called “predictive processing”) is a theory of brain function, not a cognitivist theory. From a Bayesian brain perspective, “expectations” are probabilistic predictions about the body and the world that are encoded at the level of neuronal populations. Indeed, the validity of Bayesian brain as a scientific theory rests on the actual existence of priors and PE at the neuronal level (Downey, 2018; Seymour and Mancini, 2020). However, although the Bayesian perspective does not assume that a positive expectation communicated to the patient from the clinician on a conscious level translates directly into a prior with higher precision, this cannot be excluded. In the case of pain, new research is successfully applying the Bayesian framework to the cognitive domain, exploring whether priors are translated directly at the conscious level (Mancini et al., 2021). Accordingly, it has recently been shown that humans can explicitly predict the likelihood of incoming pain intensities in a way that is consistent with Bayesian inference (i.e., not only conscious predictions were measured but also the conscious confidence of such predictions; Mancini et al., 2021). The possibility that the Bayesian brain hypothesis can extend to the description of cognitive functioning is indeed exciting, yet further research is needed before drawing such conclusions.

A crucial point, that is the second aim of this perspective, is to use these models to create clinical strategies to treat dysfunctional patients’ expectations in order to maximize treatment effectiveness, avoiding nocebo-related effects. In the following section, we offer a clinical framework for assessing and addressing MSK patients’ expectations aimed to avoid nocebo-related effects during all phases of the therapeutic encounter (i.e., history taking, physical examination, and therapeutic administration; Palese et al., 2019b; Rossettini et al., 2020a; Thomson and Rossettini, 2021).

## Discussion

### Clinical Opportunities

**Figure 2:**
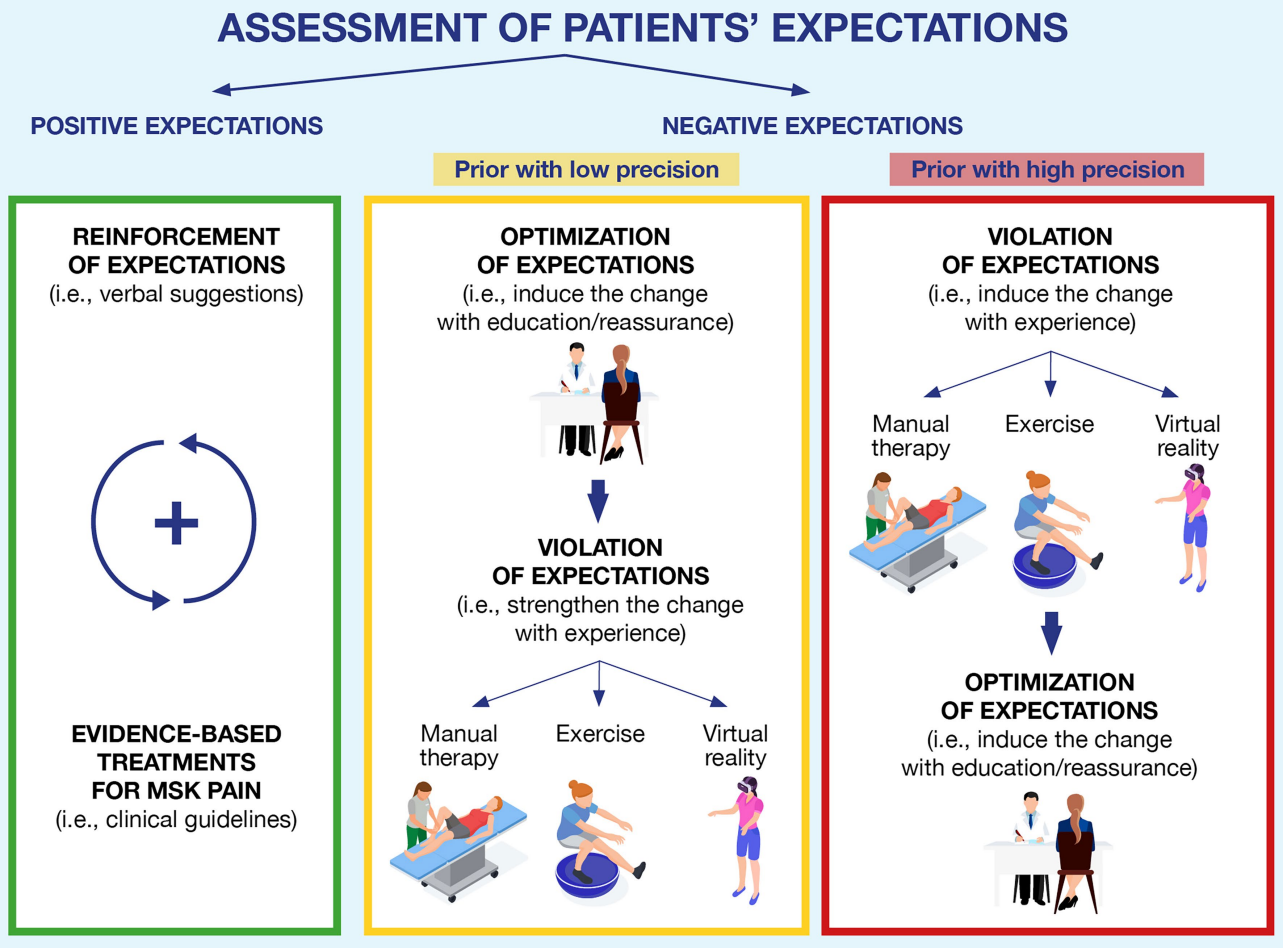
A clinical framework to assess and address patients’ expectations in musculoskeletal pain. The figure depicts the three typical scenarios that can occur in clinical practice. Green block: the patient shows positive expectations toward the rehabilitation process, and the clinician can reinforce them by providing verbal suggestions and confirming such expectations with the recommended evidence-based treatments; Yellow block: if negative expectations are detected during the initial assessment, but they have low precision, the clinician can optimize them through education and reassurance and subsequently try to violate such expectations by exposing patients to experience (using manual therapy, exercise, virtual reality, or a combination of them); and Red block: if high-precision expectations are detected, we suggest starting by exposing patients to experiences that could challenge patients’ expectations, and if this outcome is achieved, it can be reinforced through verbal suggestions (education or reassurance).

Since expectations and priors can critically change patients’ perception and adherence to a clinical treatment, their assessment and management are crucial steps in the clinical practice (Figure 2).

In the clinical encounter, clinicians can start using open questions (i.e., *“What do you expect from this therapy?”; “How do you expect the course of this condition will be?”*; Laferton et al., 2017; Rossettini et al., 2018) or specific questionnaires self-completed by the patients (i.e., the EXPECT Questionnaire, the Expectation for Treatment Scale; Jones et al., 2016; Barth et al., 2019) to assess both the direction (i.e., positive or negative) of expectations and the strength of the patients’ priors (i.e., high or low precision). This first step is important because depending on patients’ expectations, different strategies can be used. If expectations are positive, the clinician should reinforce them through verbal suggestions associated with evidence-based treatments for the MSK pain (Rossettini et al., 2018). On the other hand, if the expectations are negative, the clinician has two different strategies to address them: optimization or violation (Peerdeman et al., 2016; Kube et al., 2018).

In MSK pain, patient education and reassurance are examples of optimizations (Louw et al., 2016; Bulow et al., 2021), adopted in clinical settings, to provide information, reconceptualize beliefs and facilitate patients’ ability to cope with their condition (Watson et al., 2019). Instead, manual therapy (i.e., mobilization with movement and symptom modification procedure), therapeutic exercise (i.e., active range of motion tasks), and virtual reality (i.e., immersive scenario; Geneen et al., 2017; Gumaa and Rehan Youssef, 2019; Ahern et al., 2020; Bordeleau et al., 2021; Brea-Gomez et al., 2021; Satpute et al., 2021; Tsokanos et al., 2021) are examples of successful violation strategies commonly adopted to reduce pain and disability (Zusman, 2013a,b; Rabey et al., 2017; Bialosky et al., 2018; Geri et al., 2019; Cerritelli et al., 2021). From a Bayesian perspective, both optimization and violation can be considered as bottom-up inputs that clinicians can offer to patients to challenge the negative expectations of the patient, facilitating their updating (Friston, 2012). While optimization aims to modify one’s priors by working at a high cognitive level (i.e., providing a new understanding of the pain by explaining how it works; Doering et al., 2018), violation strategies challenge one’s dysfunctional beliefs by providing first-hand disconfirmatory evidence with experience (Craske et al., 2018), which in turn can be used to update the negative priors. Since there are currently no criteria to guide the clinicians on which strategy to use first (optimization first or violation first; Peerdeman et al., 2016; Kube et al., 2018), we suggest a clinically oriented choice which should depend on the level of precision of patients’ priors (as assessed during the clinical encounter).

Let us consider a patient that is scared of squatting because they have once read on social media that squatting repeatedly can ruin the cartilage of their knee. The clinician assesses the strength of patient’s expectations (i.e., the clinician discovers that the patient is aware that social media are full of fake news and is aware that the information about squatting and cartilage damage might not be true) and establishes that the negative expectations are likely to have low precision. In this case, we suggest that clinicians could use optimization strategies first (i.e., reducing unrealistic beliefs about possible side effects through education; Doering et al., 2018) and then violation strategies (Craske et al., 2018). By doing so, expectations are first challenged at the cognitive level *via* optimization, while violation is used at a later stage to further challenge dysfunctional expectations with experience. Since patients’ expectations are not so rooted, we would expect them to update easily, resulting in observable positive changes sooner rather than later (i.e., within a session or after a reduced number of treatments).

Instead, consider patients having negative expectations with high precision (i.e., believing that their cervical disk herniation is a severe condition limiting all neck movements). When clinicians understand that such negative expectations are firmly rooted in the patients’ minds (i.e., the patient that knows, mainly through word-of-mouth, several people whose herniation got worse because they were too active and sporty, and is therefore convinced that movement is bad for this type of condition), we suggest inverting the two strategies (violation-optimization) to avoid ruining the therapeutic alliance. If clinicians insist on telling patients information that goes against their strong expectations (i.e., optimization), patients might start losing trust in the clinicians—in other words, telling patients things that they do not want to hear can be counterproductive (Laferton et al., 2017; Rossettini et al., 2018). Instead, we propose focusing on building trust (i.e., listening to the patient, without, at first, saying things that are directly in contrast with their view), meanwhile using violation strategies so that patients can disproof their expectations for themselves (e.g., providing pain-free experiences with manual therapy, exercise, and virtual reality). Later, when patients are more open to hearing information against their initial beliefs, clinicians can use optimization strategies to further promote and strengthen priors updating (Craske et al., 2018; Schemer et al., 2019). Worth mentioning is that in the case of firmly rooted expectations (i.e., where the patient implements strategies such as cognitive immunization), patients might require more evidence before successfully updating their priors. In this situation, clinicians should consider offering a higher number of disconfirming trials (i.e., repeating violation strategies more times than usual or offering the patient more treatment sessions; Gatzounis et al., 2021; Hilleke et al., 2021).

### Challenges and Future Directions

Despite some preliminary research suggests that the Bayesian framework (Owens et al., 2018; Ongaro and Kaptchuk, 2019; Kaptchuk et al., 2020) and the ViolEx model (Kube et al., 2020; Panitz et al., 2021) are good fit for describing pain processing and symptoms persistence, some open questions remained unresolved.

First, it is crucial to understand *how clinicians can translate the strategies used to modulate patient’s expectations within the specific context of MSK pain* (Caneiro et al., 2021). So far, researchers have investigated the modulation of expectations mainly in mental health (i.e., anxiety and depression) or medical conditions (i.e., cancer and coronary heart disease; Peerdeman et al., 2016; Kube et al., 2018), compared to MSK pain (Barth et al., 2021). Therefore, the interplay between direct experiences (i.e., previous healthcare exposures), social and cultural influences (i.e., peers and media), individual differences (i.e., personality traits and genetic factor), and expectations (Panitz et al., 2021) in patients presenting different MSK pain mechanism (i.e., nociceptive, neuropathic, and nociplastic) represents a challenge for future studies (Rossettini and Testa, 2018).

Second, it is crucial to investigate if it is *possible to change patient’s expectations permanently*. Even if expectations can change in a specific situation (i.e., “*I did not feel pain in my back when bending over on this occasion*”), this modification does not necessarily translate into a general and long-lasting change (i.e., “*Every time I will bend over, I will not feel pain in the back*”; Schemer et al., 2019; Riecke et al., 2020). Furthermore, if the patient has negative expectations with very high priors, it could be difficult to change them quickly (Kube et al., 2020). Therefore, we need future studies to investigate if expectations can change for long periods (Bromberg-Martin and Sharot, 2020; Camerone et al., 2021a,b) and if they are generalized to different MSK pain conditions.

Third, it is necessary to understand *the optimal PE magnitude needed to update patient expectations*. According to the recent scientific literature, patients ignore very small PEs and avoid to update their expectations as often the cognitive costs of the change outweigh the benefits (Linton et al., 2012; Panitz et al., 2021; Pinquart et al., 2021). Furthermore, even substantial PE can be considered an exception to the rule and thus discarded without changing expectations (Linton et al., 2012; Panitz et al., 2021; Pinquart et al., 2021). Therefore, future studies should identify the magnitude of PE capable to challenge the patients’ negative expectations in MSK pain.

## Conclusion

Managing patients’ expectations continues to represent a challenge in MSK pain. Clinicians should choose wisely if, when and how to challenge patients’ negative expectations, considering whether the benefits of avoiding nocebo-related effects outweigh the risks of eroding the therapeutic alliance and having drop-outs. Based on the theoretical frameworks here presented (ViolEx Model and Bayesian Brain Hypothesis), we suggest that clinicians could use the strength of patients’ expectations as an indicator to decide when to directly challenge patients’ negative expectations (i.e., optimization), or when to start by challenging their beliefs indirectly (i.e., violation), avoiding damages to the therapeutic alliance.

## Data Availability Statement

The original contributions presented in the study are included in the article/supplementary material, further inquiries can be directed to the corresponding author.

## Author Contributions

GR, AC and MT designed and supervised the writing of the paper. GR, AC, ECam, ECar, MMan, MMir, AP and MT participated in drafting and revising the different versions of the article. All authors contributed to the article and approved the submitted version.

## Conflict of Interest

GR leads education programs on placebo, nocebo effects and contextual factors in healthcare to under- and post-graduate students along with private CPD courses.

The remaining authors declare that the research was conducted in the absence of any commercial or financial relationships that could be construed as a potential conflict of interest.

## Publisher’s Note

All claims expressed in this article are solely those of the authors and do not necessarily represent those of their affiliated organizations, or those of the publisher, the editors and the reviewers. Any product that may be evaluated in this article, or claim that may be made by its manufacturer, is not guaranteed or endorsed by the publisher.
